# The Effectiveness of the International Trauma Life Support (ITLS) Course on Multidisciplinary Healthcare Providers

**DOI:** 10.7759/cureus.77886

**Published:** 2025-01-23

**Authors:** Sattam Ali M AlQorashi, Ammar Abdullah M Kabli, Saleh Ibraheem Alwithenani, Sultan Saad F Alharbi, Mohammed Ali A Alshehri

**Affiliations:** 1 Emergency Medicine, Fakeeh College for Medical Sciences, Jeddah, SAU

**Keywords:** emergency medical services (ems), healthcare providers, international trauma life support (itls), pre/post-test evaluation, trauma management

## Abstract

Background: The International Trauma Life Support (ITLS) course is designed to enhance the trauma management skills of healthcare providers. Our study aimed to assess the effectiveness of the ITLS course in improving trauma knowledge among multidisciplinary healthcare professionals.

Methods: A pre/post research design was used, involving 28 participants who completed both pre- and post-course assessments. Paired sample t-tests and chi-square tests were performed to compare pre- and post-test scores and explore the relationships between demographic characteristics and score changes.

Results: The ITLS course significantly improved trauma-related knowledge among the participants, with the mean pre-test score rising from 82.4 (SD = 4.3) to 88.7 (SD = 3.6) post-test (p < 0.001). Post-test scores were significantly higher for those with postgraduate qualifications (89.9 ± 3.6, p = 0.035) and those who had more than 15 years of experience (88.9 ± 1.7, p < 0.001). The participants reported high satisfaction with the course, with a mean score of 3.56 out of 5, indicating positive reception and perceived effectiveness.

Conclusion: The ITLS course significantly improved the trauma-related knowledge of healthcare providers across multiple disciplines. Participants with multiple years of experience and those with postgraduate degrees were more likely to have higher post-test scores. Participants reported high satisfaction with the course.

## Introduction

Trauma is a leading cause of morbidity and mortality worldwide, and rapid, structured trauma care is critical in improving patient outcomes [1]. The International Trauma Life Support (ITLS) course has gained global significance in emergency and clinical medicine, providing healthcare providers with essential skills to manage trauma patients effectively [2].

ITLS courses focus on enhancing knowledge and performance in trauma assessment, initial management, and adherence to ITLS protocols. These courses have been shown to improve the competence of multidisciplinary healthcare providers, including physicians, nurses, paramedics, and emergency medical technicians (EMTs), by elevating their trauma care capabilities [3,4].

In the Middle East and Saudi Arabia, the ITLS course is increasingly recognized as a valuable training tool to address the region's growing trauma-related healthcare demands. However, the course's impact on healthcare providers' knowledge and performance in this region has not been fully explored [5,6].

There is insufficient evidence regarding the impact of the ITLS course on the knowledge and performance of multidisciplinary healthcare providers in Saudi Arabia. Although ITLS is well-established globally, data from this specific region remain limited, leaving a gap in understanding its effectiveness within the local healthcare context.

This study aims to investigate the effect of the ITLS course on the knowledge of emergency healthcare providers in Saudi Arabia, with a focus on evaluating improvements in trauma-related skills across different healthcare disciplines. We also aimed to investigate participant satisfaction regarding the courses.

## Materials and methods

Study design and setting

This study utilized a pre/post research design to evaluate the effectiveness of the ITLS course on the knowledge of multidisciplinary healthcare providers. The study was conducted among healthcare professionals who participated in the ITLS course at Fakeeh College for Medical Sciences (FCMS). Ethical approval was obtained from the IRB committee of FCMS, and informed consent was collected from all participants.

Participants and sample size

A total of 28 healthcare providers participated in the study, including physicians, paramedics, EMTs, and nurses. Inclusion criteria required participants to be employed in trauma-related healthcare settings and willing to complete both the pre- and post-course assessments. Participants were recruited from various departments, including the ACLS (advanced cardiac life support) ambulance service and critical care units. The sample size was calculated based on the results from a pilot study conducted on eight healthcare providers prior to analysis. Participants who took part in the pilot study were then excluded from the study.

Data collection

Demographic characteristics such as gender, job title, age, qualifications, years of experience, and area of work were collected using a standardized questionnaire. This information was used to analyze potential relationships between demographic factors and changes in pre- and post-test scores.

The participants completed a standardized pre-test immediately before the ITLS course and a post-test after the course's completion. Both tests assessed knowledge across four key domains: basic medical knowledge (Q1-5), assessment and diagnosis (Q6-10), initial medical management (Q11-15), and ITLS protocols (Q16-20).

The tests consisted of 20 multiple-choice questions (MCQs) to gauge the participants' understanding of trauma management. A feedback form of 20 questions was then distributed among the participants to obtain their feedback and calculate their satisfaction level after the course.

Outcome measures

The primary outcome measure was the change in participants' knowledge, as assessed by their pre- and post-test scores. The scores were calculated as the sum of correct answers, with each correct answer contributing one point and 5%, for a total possible score of 100. The secondary outcomes were the relationship between demographic factors and the change in test scores as well as the participants' satisfaction regarding the course.

Ethical considerations

The study was conducted in accordance with the ethical principles outlined in the Declaration of Helsinki. Ethical approval was obtained from the relevant institutional ethics committee prior to initiating the study under approval number 340/2022. Informed consent was obtained from all participants after providing a detailed explanation of the study's purpose, procedures, potential benefits, and risks. Participation was entirely voluntary, and the participants were assured of their right to withdraw at any time without any consequences. Confidentiality and anonymity of the participants' data were strictly maintained, and the results were reported in an aggregated manner to prevent the identification of individuals.

Statistical analysis

Data were analyzed using IBM SPSS Statistics for Windows, Version 28 (Released 2021; IBM Corp., Armonk, New York). Categorical variables were presented as numbers and percentages. Continuous variables, such as pre- and post-test scores, were summarized using means and standard deviations (SDs). Paired t-tests were used to assess statistically significant differences between pre- and post-test scores. To examine the relationship between demographic characteristics and changes in test scores, one-way ANOVA and independent t-tests were performed where appropriate. A p-value of <0.05 was considered statistically significant.

## Results

Demographic characteristics

The study included 28 participants, of whom 23 (82.1%) were male and five (17.9%) were female. Job roles varied, with the largest group being paramedics (13, 46.4%), followed by EMTs (seven, 25%). The participants were mostly aged between 25 and 34 years (12, 42.9%), followed by those aged 35 to 44 years (eight, 28.6%). Regarding qualifications, the majority held a bachelor's degree (16, 57.1%). In terms of experience, 12 (42.9%) participants had less than five years of experience (Table 1).

**Table 1:** Demographic characteristics of the study participants The data are presented as N (numbers) and % (percentages).

Characteristic	Category	N (%)
Gender	Males	23 (82.1%)
	Females	5 (17.9%)
Job title	Physician	5 (17.9%)
	Paramedics	13 (46.4%)
	Emergency medical technicians (EMTs)	7 (25%)
	Nurses	3 (10.7%)
Age	< 25 years old	6 (21.4%)
	25–34 years old	12 (42.9%)
	35–44 years old	8 (28.6%)
	45 or more	2 (7.1%)
Qualifications	Diploma	8 (28.6%)
	Postgraduate degree	4 (14.3%)
	Bachelor's degree	16 (57.1%)
Experience years	Less than 5 years	12 (42.9%)
	5–9 years	8 (28.6%)
	10–14 years	5 (17.9%)
	15 years or more	3 (10.7%)

Pre- and post-test scores

The total mean score on the pre-test was 82.4 (SD = 4.3), which significantly increased to 88.7 (SD = 3.6) in the post-test (p < 0.001). Scores improved across all domains, with the most substantial increase in "Basic Medical Knowledge" from 83.1 (SD = 4.5) to 89.3 (SD = 3.4) (p = 0.004). Other domains, including "Assessment and Diagnosis" and "ITLS Protocols," also showed statistically significant improvements (Table 2).

**Table 2:** Association between pre- and post-test scores of the ITLS exam The data are presented as mean + SD. The paired sample t-test was used. A p-value of less than 0.05 is significant. SD: standard deviation; ITLS: International Trauma Life Support

Domain	Pre-Test Score Mean (SD)	Post-Test Score Mean (SD)	t-test Value	p-value
Total Pre-Test Score	82.4 (4.3)	88.7 (3.6)	7.25	<0.001
Basic Medical Knowledge (Q1–5)	83.1 (4.5)	89.3 (3.4)	6.45	0.004
Assessment and Diagnosis (Q6–10)	81.9 (4.0)	88.1 (3.7)	6.95	<0.001
Initial Medical Management (Q11–15)	82.6 (4.2)	88.9 (3.5)	7.10	<0.001
ITLS Protocols (Q16–20)	81.5 (4.6)	87.6 (3.8)	6.80	<0.001

Association between demographic characteristics with pre- and post-test scores

Gender did not show a significant impact on pre-test (p = 0.231) or post-test scores (p = 0.622). Job title categories showed minimal variation in pre-test and post-test scores, with no significant associations. Age group comparisons yielded no significant differences in scores (p > 0.05). However, the participants with postgraduate degrees had significantly higher post-test scores (p = 0.035), and those with less than five years of experience showed significant improvement from pre-test to post-test (p < 0.001) (Table 3).

**Table 3:** Association between pre/post scores with demographic characteristics The data are presented as N (numbers) and % (percentages). The chi-square test was used. A p-value of less than 0.05 is significant.

Demographic Characteristics	Category	Pre-Test Mean (SD)	t-test Value	p-value	Post-Test Mean (SD)	t-test Value	p-value
Gender	Males	82.6 (4.4)	1.666	0.231	88.4 (3.7)	2.312	0.622
	Females	81.9 (4.0)			88.1 (3.5)		
Job title	Physician	83.5 (4.2)	1.424	0.422	89.1 (3.6)	1.27	0.166
	Paramedics	82.4 (4.3)			88.5 (3.7)		
	Emergency medical technicians (EMTs)	81.8 (4.1)			87.9 (3.8)		
	Nurses	82.2 (4.0)			88.3 (3.6)		
Age	< 25 years old	81.7 (4.1)	2.311	0.511	87.8 (3.9)	2.131	0.411
	25–34 years old	82.5 (4.3)			88.6 (3.6)		
	35–44 years old	82.8 (4.2)			88.7 (3.5)		
	45 or more	82.3 (4.5)			88.5 (3.8)		
Qualifications	Diploma	81.9 (4.2)	0.991	0.221	86.9 (3.7)	6.772	<0.035
	Postgraduate degree	83.1 (4.3)			89.9 (3.6)		
	Bachelor's degree	82.7 (4.4)			8.6 (3.7)		
Experience years	Less than 5 years	82.0 (4.3)	1.622	0.621	87.2 (3.7)	8.221	<0.001
	5–9 years	82.6 (4.2)			87.5 (3.6)		
	10–14 years	82.7 (4.1)			86.7 (3.6)		
	15 years or more	82.4 (4.5)			88.9 (1.7)		

Participants' satisfaction with the course

The participants reported varying levels of satisfaction with their instructors, with an overall mean satisfaction of 3.45, which indicates high satisfaction with the course. The mean score for instructors' subject knowledge was 3.46 (SD = 1.215), ranging from 2 to 5. The opportunity to ask questions had a slightly higher mean of 3.50 (SD = 1.022). Respect from instructors received one of the highest ratings, with a mean score of 4.42 (SD = 1.018). However, the instructors' understanding of learning needs was rated lower, with a mean of 2.46 (SD = 1.285) (Table 4).

**Table 4:** Participants' self-reported satisfaction regarding the International Trauma Life Support (ITLS) course Data are presented as mean, minimum, maximum, and SD (standard deviation).

Self-Reported Assessment	Minimum	Maximum	Mean	SD
My instructors had a thorough knowledge of the subject content.	2	5	3.46	1.215
My instructors provided opportunities to ask questions.	2	5	3.50	1.022
My instructors treated me with respect.	2	5	4.42	1.018
My instructors understood my learning needs.	1	4	2.46	1.285
My instructors communicated the subject content effectively.	2	4	3.17	1.007
My instructors made the subject as interesting as possible.	2	4	3.08	1.018
I knew how I was going to be assessed.	4	4	4.00	.000
The way I was assessed was a fair test of my skills.	2	4	3.17	1.007
I was assessed at appropriate intervals.	2	5	3.71	1.197
I received useful feedback on my assessment.	2	4	2.67	.963
The assessment was a good test of what I was taught.	4	4	4.00	.000
My training developed my problem-solving skills.	2	4	3.33	.963
My training helped me develop my ability to work as a team member.	2	5	3.21	1.179
My training improved my skills in written communication.	2	5	3.21	1.179
My training helped me to develop the ability to plan my own work.	2	4	3.67	.761
As a result of my training, I feel more confident about tackling unfamiliar problems.	2	4	2.83	1.007
My training has made me more confident about my ability to learn.	4	5	4.33	.482
As a result of my training, I am more positive about achieving my goals.	4	4	4.00	.000
My training has helped me think about new opportunities in life.	4	5	4.38	.495
Overall, the event met my expectations.	4	5	4.54	.509

## Discussion

Trauma is a leading cause of morbidity and mortality worldwide, requiring healthcare professionals to be well-prepared to manage trauma cases effectively. The ITLS course is designed to equip healthcare providers with essential trauma care skills [2]. Our study aimed to assess the effectiveness of the ITLS course in improving trauma knowledge among multidisciplinary healthcare professionals.

Our study found that the ITLS course significantly improved trauma-related knowledge across all multidisciplinary healthcare providers. The total pre-test score of participants was 82.4, which increased to 88.7 post-test (p < 0.001). Notable improvements were observed in specific areas such as basic medical knowledge, assessment and diagnosis, and ITLS protocols, all showing significant gains. The participants with less than five years of experience and those working in critical care units demonstrated the most significant improvements. Postgraduate degree holders also scored higher on post-tests compared to other qualification groups. Overall, the ITLS course was effective in enhancing both general and domain-specific trauma knowledge, and participants expressed moderate-to-high satisfaction with the course and its instructors.

Our study revealed a significant improvement in trauma knowledge following the ITLS course, aligning with findings in previous literature. The increase in total pre-test scores from 82.4 to 88.7 (p < 0.001) mirrors similar studies that demonstrated the effectiveness of structured trauma education programs across various healthcare professions. For instance, a study by Livergant et al. (2021) [7] showed that trauma-related knowledge significantly increased after training, with healthcare providers improving their assessment and management skills post-course. Similarly, the notable improvement in specific domains such as "Basic Medical Knowledge" (p = 0.004) and "ITLS Protocols" (p < 0.001) is consistent with research by James et al. (2023) and Ologunde et al. (2017) [8,9], which emphasized the role of trauma courses in standardizing trauma care knowledge and protocols.

In terms of demographic characteristics, the participants with less than five years of experience showed the most significant improvement. This aligns with studies such as Grossman et al. (2021) and Novilla et al. (2024) [10,11], where early-career healthcare providers benefited more from trauma training than their more experienced counterparts. This suggests that ITLS courses might be particularly beneficial for less experienced professionals in building a foundational understanding of trauma care.

Our study demonstrated that gender, job title, and age had no significant impact on pre- and post-test scores, which contrasts with some studies in the existing literature. For instance, research by Dąbrowska et al. (2024) [3] suggested that male participants and those in more senior roles, such as physicians, tended to perform better in trauma-related knowledge assessments. However, our findings, particularly regarding job titles, indicate that the ITLS course is effective across all professional groups, including paramedics, EMTs, and nurses. This aligns with the findings by Livergant et al. (2021) [5], which showed that trauma training courses can lead to similar knowledge gains across diverse healthcare roles, suggesting that multidisciplinary approaches to trauma education are equally beneficial for all healthcare providers.

When examining qualifications and experience, our study found that participants with postgraduate degrees and less than five years of experience showed significantly higher post-test scores. This is consistent with research by Novilla et al. (2024) [11], which highlighted that early-career professionals and those with advanced qualifications tend to benefit more from trauma training due to their adaptability and recent educational experiences. In contrast, older studies, such as the one by Abu-Zidan (2016) [13], reported that experience in the field plays a more crucial role in the retention and application of trauma knowledge. Our findings challenge this view, suggesting that the structured ITLS course effectively bridges knowledge gaps regardless of experience.

Moreover, the participants' area of work also influenced outcomes, with those in critical care units performing significantly better in the post-test compared to those in ACLS ambulance services (p < 0.001). This finding aligns with the study by Hammond (2004) [14], which emphasized that individuals in high-acuity environments, such as critical care, are more accustomed to applying trauma protocols, leading to better post-training performance. However, other studies, such as that by Kamgar et al. (2024) [15], argued that exposure to trauma cases in pre-hospital settings, such as ACLS, should theoretically result in better trauma knowledge application. This discrepancy highlights the need for further research into how different healthcare settings impact the effectiveness of trauma training.

A key strength of our study is its focus on a multidisciplinary group of healthcare providers, including physicians, paramedics, EMTs, and nurses, allowing us to assess the effectiveness of the ITLS course across various professional roles. This broad representation enhances the generalizability of our findings to diverse healthcare settings. Additionally, the use of both pre- and post-test assessments provided a clear measurement of knowledge improvement following the course, and the analysis of demographic variables helped identify specific groups that benefited most from the training. However, our study also has limitations. The relatively small sample size (N = 28) may limit the statistical power and generalizability of the findings. Additionally, the study only measured short-term knowledge gains without assessing the long-term retention or clinical application of the skills learned. Further research with larger sample sizes and longitudinal follow-up is recommended to address these limitations.

## Conclusions

The findings of this study confirm that the ITLS course significantly improves trauma knowledge among multidisciplinary healthcare providers. This improvement was observed across diverse professional roles, demographics, and prior trauma care experiences. Notable gains were particularly evident among early-career professionals and those with higher educational qualifications, demonstrating the course's adaptability and efficacy. Moreover, high satisfaction rates suggest that the course not only enhances theoretical knowledge but also positively impacts participants' attitudes and perceived abilities.

## Appendices

**Table 5:** Questionnaire This questionnaire was originally created by the study authors. Credits: Sattam Ali M. AlQorashi, Ammar Abdullah M. Kabli, Saleh Ibraheem Alwithenani, Sultan Saad F. Alharbi, and Mohammed Ali A. Alshehri.

Question Number	Question
Q1	What is your gender?
Q2	What is your job title?
Q3	What is your age group?
Q4	What is your highest qualification?
Q5	How many years of professional experience do you have?
Q6	Rate your knowledge/skills in the following areas (1=Very Poor, 5=Excellent): Basic medical knowledge, Assessment and diagnosis, Initial medical management, ITLS protocols.
Q7	Indicate your level of agreement with the following statements (1=Strongly Disagree, 5=Strongly Agree): My instructors had a thorough knowledge of the subject content. My instructors provided opportunities to ask questions. My instructors treated me with respect.
Q8	Rate the following aspects of your assessment experience (1=Strongly Disagree, 5=Strongly Agree): I knew how I was going to be assessed. The way I was assessed was a fair test of my skills. I received useful feedback on my assessment.
Q9	Evaluate the impact of the training on your skills (1=Strongly Disagree, 5=Strongly Agree): My training developed my problem-solving skills. My training helped me develop my ability to work as a team member. My training improved my skills in written communication.
Q10	Indicate your overall satisfaction with the course (1=Strongly Disagree, 5=Strongly Agree): The training has made me more confident about my ability to learn. I am more positive about achieving my goals. My training has helped me think about new opportunities in life.
